# Cognitive Individual Differences in Multilingualism: Language Aptitude and Working Memory in L3 Learners

**DOI:** 10.1007/s10936-026-10268-3

**Published:** 2026-07-01

**Authors:** Elifcan Öztekin, Gülcan Erçetin

**Affiliations:** Department of Foreign Language Education, Boğaziçi University, 34342 Bebek, Istanbul, Türkiye

**Keywords:** Language aptitude, Working memory, L3 learning, L3 listening and reading comprehension

## Abstract

This study investigated the relationships of language aptitude (LA) with working memory (WM) and their predictive roles in explaining third language (L3) comprehension. A sample of L1 Turkish-speaking English language teaching majors (*N* = 109) completed the LLAMA version 3 (Meara & Rogers, 2019), forward digit span, rotation, symmetry, and operation span tasks (Foster et al., 2015). L3 listening and reading comprehension measures were also administered to a subgroup of participants with L3 learning experience (*N* = 33). A factor analysis indicated verbal/phonological memory (digit span, operation span, and partially LLAMA D), visuospatial WM (symmetry and rotation span), and LLAMA (LLAMA B, E, F, partially D) factors. The L3 experience was not significantly correlated with any of the cognitive factors. The cognitive factors did not significantly contribute beyond L3 experience in explaining variance in L3 reading and listening comprehension. Findings were discussed regarding the verbal abilities shared by LA and WM and the potential sources of variation within L3 experience in young adult populations.

**Supplementary Information:**

The online version contains supplementary material available at 10.1007/s10936-026-10268-3.

## Introduction

Cognitive individual differences research in the second/foreign language (L2) learning field involved working memory (WM) and language aptitude (LA) as two main factors, closely related to each other in terms of the memory and processing functions they are assumed to tap in the related theories. While both WM and LA were observed to correlate with several aspects of language such as grammar, vocabulary, and language skills on their terms, their interaction has not been resolved yet, with the recent theoretical elaborations suggesting potential outlines of such an interaction to be probed with empirical evidence (e.g., Wen & Skehan, 2021). The rise of multilingualism through the ubiquitous contexts of learning additional foreign languages in today’s world makes understanding the potential implications of multiple language learning experiences on cognitive factors a significant research problem. The literature on the cognitive advantages of bilingualism (e.g., Bialystok, 2009, 2011) is extended to third or more language (L3/n) learning contexts. The “bilingualism effect” can be described as the enhanced executive functions of bilingual individuals compared to monolingual counterparts due to the inherent task of managing the activation of more than one language in their cognitive system. (e.g., Green & Abutalebi, 2013). The multilingualism extensions of the bilingualism effect have reported mixed findings so far (e.g., Guðmundsdóttir & Lesk, 2019; Ma et al., 2022; Madrazo & Bernardo, 2018). Additionally, studies particularly investigating L3/n effects on complex WM and LA components report that if observed, these effects interacted with factors such as perceived multilingualism (e.g., Thompson, 2013), balanced language proficiency, socioeconomic status or general intelligence (e.g., Cockcroft, 2024; Espi-Sanchis & Cockcroft, 2022).

Regarding the current state of the related research, the present study aims to investigate the relationship between the cognitive individual factors and extended foreign language learning experience. To this end, the cognitive abilities tapped by the LA and WM are examined in an additional foreign language learning context with young adult undergraduate students at an English-medium university in Turkiye. This objective is staged in three levels. First, the study explores the construct-level structures of LA and WM to establish a methodological basis to represent these constructs as measured with frequently reported instruments in the literature, i.e., the LLAMA tests for foreign language LA and verbal and visuospatial WM span tests. Second, it probes the relationship between multilingual experience and these cognitive factors of LA and WM in an additional foreign language learning context to test whether the amount of additional language learning experience beyond extensive L2 instruction is associated with differences in LA and WM And third, it investigates the predictive roles of LA and WM factors on listening and reading comprehension in the additional foreign language in a smaller sample of participants to observe whether individual differences in LA and WM explain any variance in the outcomes of additional foreign language beyond multilingual experience per se. This study represents multilingual experience on a continuous scale accounting for time the participants spent learning an additional language beyond their L2 English in developing proficiency levels defined as “L3 experience”. In a smaller scale, the predictive roles of LA and WM factors on L3 listening and reading comprehension are also probed beyond this time-oriented L3 experience in a smaller number of participants who studied L3s in which listening and reading comprehension tests were available in a common framework. This design provides a variation to the multilingual LA and WM studies presenting comparisons based on the number of languages studied (e.g., Ma et al., 2022; Huang et al., 2022) by presenting a time-based variable of L3 instruction i.e., the number of courses completed in a row increasing in level of proficiency in one particular L3. It also aims to contribute to the body of research on the cognitive individual difference factors (i.e., LA and WM) with relation to extended experience of L3/n instruction beyond an L2 in young adults at university level. Findings could help observe if LA and WM abilities measured by these instruments relate and if so, what sort of patterns exist in the presence of an additional L3 as an extension of L2 studies of LA and WM.

## Background Literature

### Language Aptitude and Working Memory in L2 Learning Research

LA became an increasingly investigated cognitive individual difference, presenting an accumulation of empirical research probing its role in second/foreign language learning contexts (e.g., Li, 2015, 2016; Wen et al., 2017). While there were revisits of its theoretical definitions (e.g., Skehan, 2016, 2019) and relationships with other cognitive factors such as memory (e.g., Carroll, 1990), LA was observed to correlate with second/foreign language abilities such as vocabulary and grammar learning (e.g., Erlam, 2005; Robinson, 1997, 2002; Yalçın & Spada, 2016), the development of the four skills (e.g., Granena, 2019; Saito, 2017) or overall L2 proficiency (e.g., Artieda & Munoz, 2016).

Simply defined as the limited capacity of storage and processing, WM was frequently addressed in language studies mostly due to its elaborated emphasis on verbal/phonological processes relating to the role of WM in language development. Baddeley’s (2000) latest revisited model of WM included two slave systems (i.e., the phonological loop and visuospatial sketchpad) managed by a central executive and an episodic buffer allowing interaction between the two slave systems. However, more compact descriptions of WM defined it as a function of attention at the temporarily conscious level rather than a staged system initiated at the short-term storage capacity (e.g., Engle, 2002; Engle & Kane, 2004; Engle et al., 1999). WM entailed the managerial functions of executive abilities such as suppressing distractions or switching between tasks at a highly activated conscious attention level. Shah and Miyake (1996) named these different accounts of WM as “unitary vs. non-unitary camps” of WM, emphasising the componential and compact natures of the two approaches. Although the two approaches differed in the frames of the construct, the functions and abilities attributed were still compatible with each other (Dehn, 2008).

The measurement of WM mainly included simple and complex tasks, differentiating between the storage and processing capacities (Conway et al., 2005). Simple tasks measured the storage capacity such as digit or letter span tasks, while complex tasks involved simultaneous processing and storage abilities such as operation and reading span tasks. Research on the role of WM in L2 learning presented that the complex measures of WM were more closely related to L2 functions such as reading comprehension rather than simple storage tasks (e.g., Daneman & Merikle, 1996) as well as an overall relationship L2 grammar (e.g., Williams & Lovatt, 2003) and vocabulary learning (e.g., Martin & Ellis, 2012), general proficiency (e.g., Mitchell et al., 2015; Serafini & Sanz, 2016; Service et al., 2002), or skills development (e.g., Juffs & Harrington, 2011). There was a more robust positive relationship between L2 learning outcomes and verbal rather than non-verbal and processing rather than storage tasks (e.g., Çeçen & Erçetin, 2016; Linck et al., 2014).

The relationship between LA and WM in L2 learning was examined in various contexts. The complex tasks measuring storage and processing capacities of WM as opposed to simple storage tasks measuring phonological short-term memory produced different relationships with different subtests of the MLAT (e.g., Hummel, 2009; Sáfár & Kormos, 2008; Winke, 2013). The LLAMA tests (Meara, 2005) also provided such differentiation of subtests concerning other cognitive factors. Granena’s (2013) findings from a factor analysis study indicated that the three subtests of LLAMA (vocabulary learning, sound-symbol correspondence, and grammatical inferencing) loaded onto a separate factor from the sound recognition subtest, which loaded onto the factor represented with implicit learning, while operation span remained as a separate factor. This separation between the LLAMA subtests in factor analysis was observed in different studies, highlighting the implicit and explicit LA interpretation (e.g., Xiang et al., 2012; Yalçın et al., 2016).

WM was observed to be a domain-general cognitive individual factor involved in non-linguistic as well as linguistic processing functions (Redick et al., 2012). LA, on the other hand, has been defined as domain-specific abilities specialised in processing language and linguistic material. The question of whether LA lies within the structure of WM as a domain-general ability or they constitute two separate structures is still not fully answered. Empirical studies showed that they are not strongly correlated, meaning that they could represent distinct abilities (Li, 2016). However, there is also not a consistent map of the abilities composing the two constructs. At a theoretical level, the involvement of these constructs in L2 learning can be differentiated in terms of the level of consciousness on the implicit and explicit learning continuum. Implicit and explicit knowledge were defined as two types of L2 knowledge. Particularly instructed L2 learning was associated with the conscious attention level, building explicit knowledge, and the constant use of certain aspects of this knowledge might lead to “automatisation”, taking the knowledge down to the conscious attention level and reducing the explicit processing of that knowledge (e.g., DeKeyser, 1997). DeKeyser (2019) highlighted the importance of understanding and measurement of implicit L2 knowledge, due to the difficulty of tapping unconscious information without activating the explicit attention processes. Therefore, it becomes an important task to understand the cognitive processes that the LA and WM represent. Skehan (2019) emphasised the potential place of language-specific abilities even in domain-general approaches to L2 learning due to the particularities of learning a language. Wen and Skehan (2021) proposed a theoretical elaboration on possible interactions of LA and WM throughout different stages of L2 learning. These theoretical approaches also call for empirical evidence and accurate measures of the constructs.

### Multilingual Experience in Language Aptitude and Working Memory Relationship

The research findings reporting higher performance of bilingual individuals compared to their monolingual counterparts in tasks measuring executive functions (i.e., inhibitory control, task switching, updating, etc.) were associated with an advantage of bilingualism in these cognitive abilities (e.g., Bialystok, 2009, 2011). The higher performance in executive functions was attributed to the inherent task of bilinguals to manage the competing activation of two language systems by suppressing the activation in the irrelevant (Green &Abutalebi, 2013).

Studies extending these findings to learning a third or even more languages (L3/n) reported mixed results. For instance, several studies comparing trilinguals, bilinguals, and monolinguals in inhibitory control and task-switching tasks indicated that bilinguals and trilinguals outperformed their monolingual counterparts, with no substantial difference between bilinguals and trilinguals (e.g., Ma et al., 2022; Madrazo & Bernardo, 2018; Poarch & Van Hell, 2012; Schwieter & Sunderman, 2011). Furthermore, there was contradictory evidence indicating a negative effect of age in inhibitory control tasks in the trilingual group, which was not not observed in the bilingual and monolingual groups (Guðmundsdóttir & Lesk, 2019). These findings call for caution in even assuming the bilinguals and trilinguals are comparable in terms of these cognitive abilities since there could also be negative effects of additional language learning in contexts.

The mixed results from the cross-sectional multilingualism studies were observed in also within-subject designs. Bouffier et al. (2020) implemented vocabulary and picture naming tasks in the three languages of young adult speakers of L1 Luxembourgish, L2 German, and L3 French as a measure of language proficiency. The focus of attention and controlled attention involved recalling items from a series of digits and letters or locations of visual items non-selectively (focus) or selectively (controlled) while the auditory-verbal WM was measured using serial word and nonword recall tasks in the L1. The results showed a stronger relationship of the less proficient L3 to the auditory-verbal WM than the more proficient L2 and the dominant L1, in a similar line as previous WM and L2 proficiency studies (e.g., Serafini & Sanz, 2016) reporting a clearer relationship to WM in earlier levels of L2 learning at lower levels of proficiency.

Van den Noort et al. (2006) investigated the effects of language proficiency on WM across three languages. The participants were native Dutch, fluent L2 German, and beginner L3 Norwegian speakers. They completed two complex WM tasks, which included reading span in the three languages and a letter-number ordering span, as well as simple storage tasks, such as digit span. Accuracy and reaction time were measured. The findings showed that participants performed significantly better in their native language than in their L2 and L3 in complex WM tasks. They also performed better in their fluent L2 compared to their beginner L3 in the reading span task, but not in the letter-number ordering span. However, simple storage tasks showed no significant differences between the languages, except for higher accuracy in the L1 compared to the L3. The study suggests that WM is language-specific for complex functions, but not for simple storage tasks.

A concern with measuring WM through linguistic resources that rely on language proficiency is that it may not apply to diverse proficiency levels in different languages. Therefore, non-linguistic measures of WM involving verbal and visuospatial storage and processing tasks make it more applicable to measure WM with diverse proficiency levels in different languages. Cockcroft et al. (2019) implemented verbal and visuospatial storage and processing tasks from the Automated Working Memory Assessment (AWMA) battery to measure WM in young adult university students in South Africa who were proficient in three to five local South African languages and advanced in English. A control group of native English speakers was also included. The study controlled for socioeconomic status (SES) to avoid affecting language proficiency and intellectual ability. The results showed that the multilingual group outperformed the monolingual control group in both verbal and visuospatial storage and processing tasks of WM when SES and verbal intelligence scores were controlled for.

Cockcroft (2022) further investigated whether the number of languages spoken by participants had any effect on WM. The findings showed that the number of languages spoken was negatively correlated with scores in the visuospatial storage and processing tasks of WM. However, Espi-Sanchis and Cockcroft (2022) did not confirm these findings about visuospatial WM. Instead, they found that balanced proficiency across the different languages, as self-rated on the “Language Experience and Proficiency Questionnaire,” could be an important covariate since it was observed to be a significant predictor of the verbal processing component of WM.

Research in LA also points to some relationship to the experience of foreign language learning. For instance, Rogers et al. (2017) stated that individuals with instructed foreign language learning experience outperformed monolinguals in LLAMA B (vocabulary learning) and F (grammatical inferencing). Thompson (2013) also reported a positive relationship between multiple language learning experiences and CANAL-F test scores. Ma et al. (2018) compared English language majors with and without additional foreign language learning experience beyond their L2 English in a Chinese university. LLAMA subtests showed significant differences in LA between the additional foreign language experiencing group and the L2 group in vocabulary learning (LLAMA B) and sound-symbol correspondence (LLAMA E). The study aimed to investigate whether the group of L3 learners acquiring either French or Japanese language showed any difference in the LLAMA subtests. The results revealed only a slight difference in LLAMA B scores, with the L3 Japanese group performing better than the L3 French group.

There are very recent studies investigating the LA and WM interaction with multiple language learning experiences. For instance, Huang et al. (2022) examined the LLAMA and Operation span scores of simultaneous L2 English and L3 Japanese or Russian learners compared to only L2 English learners with L1 Chinese-speaking freshman and sophomore cohorts. Using a pre/post-test design measuring LA and WM before and after L2 or L2 + L3 learning experience, the study indicated some improvement in the operation span scores for both groups over one academic year. However, the L2-only group did not significantly differ from the L2 + L3 learning group in the LLAMA subtests. Only the freshman L2 + L3 group had a significantly higher WM gain score than the L2-only group. Similarly, Huang et al. (2021) investigated whether L2 English and L2 English+L3 Japanese learners in a Chinese undergraduate program differed in their LA and WM scores. The results showed significantly higher fractal 2-back task and LLAMA D (sound discrimination) scores of the L2 + L3 group than the L2-only group, while all the other LLAMA subtests were not significantly different. Hierarchical regression analyses also showed that language experience and WM significantly predicted only LLAMA D. These two empirical studies conducted in similar language and participant settings operationalising the LA and WM with similar instruments presented mixed results regarding the multiple language learning experience and the LA and WM. Therefore, the interpretation regarding the role of multilingual experience in LA and WM factors could gain reliability through empirical evidence from different contexts with alternative language combinations and experiences.

### The Present Study

This study aims to probe the relationships between visuospatial WM (Rotation Span and Symmetry Span tasks), verbal WM (Operation Span), and phonological short-term memory (PSTM, digit span) and LA abilities with LLAMA tests version 3 (Meara & Rogers, 2019) with relation to L3 domain experience (number of semesters spent taking an additional foreign language) to address the following research questions:

1. What are the structural relations between LA and WM measures?
2. Is L3 experience related to LA and WM measures?
3. Do LA and WM factors significantly predict L3 listening and reading comprehension beyond L3 experience?

#### Hypothesis 1

The subtests of LA and WM are hypothesized to create separate but correlated factors (e.g., Granena, 2013; Xiang et al., 2012; Yalçın et al., 2016). Therefore, a structural separation is predicted between the LA and WM subtests. However, no specific predictions are made regarding the factor structures since there is variation in terms of the loadings of the subtests, particularly for the LLAMA tests.

#### Hypothesis 2

Reflecting on the previous findings showing relationships of L2 proficiency with LA and WM (e.g., Artieda & Muñoz, 2016; Serafini & Sanz, 2016; Thompson, 2013), some relationship of L3 experience with LA and WM factors are expected. However, no particular directions (i.e., positive or negative relationships) are predicted.

#### Hypothesis 3

Based on previous findings in L2 research (e.g., Alptekin & Erçetin, 2011; Kara-Duman et al., 2021; Leeser, 2007; Sparks et al., 2011), some predictive roles of LA and WM factors on L3 reading and listening comprehension are expected beyond the L3 experience.

## Method

### Participants

The study presents data from 109 undergraduate English language teaching majors in an English-medium university in Turkiye. The language background involves Turkish as L1, English as high-level proficiency L2 with extensive instruction and academic use, and various languages as L3s emerging with mainly classroom instruction experience at varying proficiency levels. The sample includes participants from freshman to senior year cohorts with an overall number of 80 female and 29 male participants and an overall mean age of 21.13 (*SD* = 1.46).

The varying amounts of L3 learning experience of the sample population present an important factor, which the present study represents as “L3 experience”, following Payne et al. (2009), who define the “domain experience” as “time spent actively learning the language” (p. 119) and measure it as the total number of L2 classes taken. Payne and colleagues investigate this operational definition of domain experience to test whether L2 domain experience (i.e., time spent actively learning L2) enhances L2 comprehension for individuals already with higher WM, or domain experience acts independently from WM with relation to L2 reading comprehension. In a similar line, our study tests whether L3 domain experience interacts with cognitive factors of WM and LA, and in a smaller scale, proceeds to test this connection in L3 comprehension. This definition of L3 experience also provides a connecting thread which can be an objective scale across the wide range of different languages taught at the same unit of modern languages of one single higher education institution in the current study. Accordingly, the amount of L3 domain experience was quantified by the number of L3 classes taken in row following the graded proficiency levels at the university. Therefore, the L3 experience was coded as “1” for a participant who took the elementary 101 level of an L3, while it was coded as “3” for a participant who took the intermediate 201, since this level represents the third course in a row starting from the very first 101 course level. The additional foreign languages at the university are offered as courses in a semester of 13–14 weeks, meeting for 6 h a week. A control group with no additional foreign language course background was also included and coded as the L2-only group. A breakdown of frequencies across the levels of L3 domain experience is provided in Appendix A. The distribution of L3 experience across the different languages is also provided in Appendix B.

The participants’ L1 Turkish and L2 English were checked for overall proficiency using general vocabulary knowledge tests in these languages. The vocabulary knowledge subtests (i.e., providing synonyms, antonyms, and completing analogies) of the Woodcock-Johnson achievement test battery were administered to represent the overall L1 Turkish linguistic knowledge in the Turkish-adapted version (Erçetin et al., 2014). The English vocabulary size test (Nation & Beglar, 2007) was used to represent the overall L2 English proficiency. One-way analysis of variance on L2 English vocabulary size scores with L3 domain experience as the between-groups independent variable indicated no significant difference across the groups, *F* (3, 106) = 0.183, *p* >.05. A multivariate analysis of variance on the three subtests of the L1 Turkish vocabulary knowledge test with the L3 domain experience as the independent variable also indicated no significant differences across the four groups, *F* (3, 106) = 0.970, *p* >.05; Wilk’s Λ = 0.921.

### Instruments

#### The LLAMA Tests

The updated online versions of the four LLAMA subtests (Meara & Rogers, 2019) were implemented to measure LA, available at https://www.lognostics.co.uk/tools/LLAMA_3/. The four subtests aim to tap vocabulary learning (LLAMA B), sound recognition (LLAMA D), sound-symbol correspondence (LLAMA E), and grammatical inferencing (LLAMA F). Each subtest is scored out of 20 based on accuracy and the final score is reported upon completing the subtest on the web browser, with updates and improvements from the earlier version 2 (Rogers et al., 2023). The participants were given the test instructions in the L1 Turkish, following the LLAMA manuals. Following Rogers et al. (2017) reporting no significant difference between note-taking and no note-taking conditions, note-taking was not allowed in the LLAMA E and F subtests to balance the task difficulty and time taken to complete the tests.

#### Memory Tasks

The shortened automated versions (Foster et al., 2015) of operation (OSpan), rotation (RotSpan), and symmetry (SymSpan) span tasks were administered to measure complex WM. As non-linguistic measures of memory, the OSpan includes verbal input material (i.e., arithmetic operations for processing and letters for recall), while RotSpan and SymSpan include visuospatial input materials (i.e., mental rotation and distinguishing symmetry for processing and recalling arrows and squares for storage). Following the models that Foster et al. present to shorten the time to implement WM tasks while keeping reliable predictive power as in full versions, three blocks of SymSpan, 2 blocks of RotSpan, and one block of OSpan were implemented using the tasks available at https://englelab.gatech.edu/ in E-prime 3 script.

To probe the construct representation of the sound recognition subtest of the LLAMA, which was reported to relate to PSTM (Granena, 2013), a forward digit span as a simple storage function representing PSTM was implemented available at https://support.pstnet.com/hc/en-us/articles/360044725853-Digit-Span-Forward-Task-34456.

#### L3 Comprehension Tests

The listening and reading comprehension sections of some standard general proficiency tests in French, German, Italian and Spanish languages (see Appendix C for instrument details) were implemented with the participants studying one of these languages as their L3 in the present study to represent a sample of L3 outcome as a dependent measure. To designate a common proficiency level across the different languages with some functional language knowledge, tests for the B1 level in the Common European Framework of Reference for Languages (CEFR) were chosen to implement in all languages to all participants to measure L3 comprehension. Since language proficiency tests based on a common and comparable proficiency framework were available for these four European languages, it was possible to collect L3 comprehension data in these languages. The specific sections and the item types used are presented in Appendix C.

#### Procedure

The participants took the tasks in individual sessions with the first author in a computer lab on the university campus. The researcher introduced the project in person in some departmental courses and via email. Interested participants were invited to choose a suitable meeting slot in an online scheduling app. The total number of students approached was 218, yielding an acceptance rate of 50.91%. Participants completed a background questionnaire as an online form before the lab session. The tasks followed the following order: L1 vocabulary tests, forward digit span, complex WM tasks (OSpan, RotSpan, and SymSpan), LLAMA subtests (B, D, E, and F), L2 vocabulary test, L3 listening and reading comprehension tests.

Participants completed all tasks except L3 comprehension tests, which were given only to those studying foreign languages. A session lasted approximately 2 h 15 min, or 3 h including L3 tests. Participants took a 10-minute break after LLAMA tests if needed before L3 tests.

#### Data Analysis

As the data processor, SPSS statistical package version 27.0 was used for all calculations. The data were initially examined for univariate and multivariate distributions and the assumptions of the statistical analyses. Considering the lower range (min. 4 and max. 9) and variability (*SD* = 1.08) in digit span, nine missing cases (8.4% per cent of the entire sample) were imputed with the mean value. The missing cases in the remaining cognitive variables (missing percentages ranging between 0.09 and 3.7) were not imputed to keep the dataset intact regarding the individual differences. Accordingly, pairwise exclusion of missing cases was implemented in reporting the descriptive statistics and examining the overall correlations among the cognitive factors, while listwise exclusion was implemented in prediction analyses.

The univariate normality assumption for all variables was cross-checked visually with histograms and box plots, and statistically with Kolmogorov-Smirnov (K-S) normality tests and the skewness and kurtosis values. The variables that had skewness and kurtosis values between -/+ 1.00 range were accepted normally distributed. Log10 and square root transformations were applied to the reflected scores of OSpan and SymSpan due to violations of normality with negative skewness and leptokurtic distribution. The LLAMA test scores out of 20 were used in all the analyses. Instances of zero scores were checked within the overall subtest distributions by the -/+ 3 SD criterion and two cases of zero scores beyond − 3 SD below the mean on the LLAMA D were removed, while zero scores on the other subtests were kept since they were within the -/+ 3 SD range. Finally, the multivariate normality was checked using the Mahalanobis distance criterion of *p* <.001 across the subtests of LLAMA, digit span, and complex WM tests, observing no multivariate outliers. For the regression analyses with L3 experience predicting cognitive factors, the assumption of homoscedasticity was checked through the distribution of standardized residuals, with all *z* cores of the residuals normally distributed (all K-S test *p*s > 0.05). For the hierarchical regression analyses to predict L3 comprehension scores, both models were checked for the normality of the standardized residuals normally distributed (Shapiro-Wilk test *p*s > 0.05). The collinearity diagnostics was checked against multicollinearity in the hierarchical regression analyses with multiple predictor variables on L3 comprehension scores with all tolerance values above the 0.2 threshold (Field, 2013). The anonymised dataset file and the details of the data collection materials will be shared on the Open Science Framework (OSF) repository for this manuscript.

## Results

The descriptive statistics of the measures for the entire sample of participants are presented in Table 1. The complex WM span scores are the raw data before the data transformations. The vocabulary learning LLAMA subtest had the lowest mean accuracy while the grammatical inferencing subtest had the highest mean accuracy.

**Table 1:** Descriptive statistics for the entire sample of participants

Measures	*N*	Missing *N*	M	SD	Min	Max	Skew.	Kurt.
LLAMA B (Possible max. = 20)	108	1	9.31	4.37	0	19	0.31	− 0.57
LLAMA D (Possible max. = 20)	108	1	11.27	2.9	2	18	− 0.55	1.08
LLAMA E (Possible max. = 20)	108	1	11.30	5.06	0	20	− 0.40	− 0.86
LLAMA F (Possible max. = 20)	108	1	12.10	3.6	4	19	0.03	− 0.70
OSpan (Possible max. = 25)	107	2	20.57	4.09	7	25	−1.15	1.10
RotSpan (Possible max. = 14)	105	4	8.99	3.06	0	14	− 0.49	− 0.24
SymSpan (Possible max. = 42)	107	2	28.78	7.82	0	42	− 0.79	1.15
Digit span (Possible max. = 9)	100	9	7.09	1.08	4	9	− 0.42	0.26
L3 listening comp. percentage	35	74	55.64	14.47	26.67	90	0.18	− 0.15
L3 reading comp. percentage	35	74	58.19	15.27	30	84	− 0.09	−1.2
L3 experience	108	1	2.38	1.99	0	8	0.62	− 0.38

Pairwise deletion of missing values applied. Percentages of missing value range between 0.9% and 3.7% for the cognitive variables, and 67.9% for the L3 comprehension scores out of a total of 109 participants.

To address the first research question regarding the structural relationship between the LA and WM measures, bivariate Pearson product-moment correlations were calculated as shown in Table 2.

**Table 2:** Bivariate correlations among the LA and WM measures

	1	2	3	4	5	6	7	8
1. LLAMA B	1							
2. LLAMA D	0.206*	1						
3. LLAMA E	0.269**	0.205*	1					
4. LLAMA F	0.454**	0.184	0.348**	1				
5. OSpan	− 0.055	0.015	0.256**	0.153	1			
6. RotSpan	0.144	0.059	0.129	0.128	0.225*	1		
7. SymSpan	0.158	0.184	0.239*	0.323**	0.266**	0.487**	1	
8. Digit span	0.101	0.210*	0.075	0.148	0.260**	0.106	0.247*	1

*p <.05, **p <.01Pairwise deletion of missing values applied with *N*s displayed in Table 1

To examine the construct-level structural pattern, an exploratory factor analysis (EFA) applying the principal component technique was conducted. The descriptive statistics of the variables as they were entered in the EFA are presented in Appendix D. The sampling adequacy was confirmed with a Keiser-Meyer-Olkin (KMO) value of 0.654 above the accepted threshold of 0.50 (Field, 2013) and the sphericity assumption was met with a significant Bartlett’s test result, χ^2^ (28) = 124.39, *p* <.001. A three-factor solution with the criterion of eigenvalues greater than 1 explained 60.47% of the total variance. The three factors explained 21.95, 19.72, and 18.8% of the variance respectively. The communalities ranged between 0.435 and 0.735. To probe the factor structure by both divergence and convergence rotation methods, both varimax and direct oblimin rotation methods were conducted. The factor structure remained the same in both rotation methods. Table 3 presents the varimax-rotated factor solution.

**Table 3:** The varimax-rotated factor solution

	Factor 1	Factor 2	Factor 3
LLAMA B	0.824		
LLAMA D			0.552
LLAMA E	0.541		
LLAMA F	0.760		
OSpan		0.419	− 0.633
Rotspan		− 0.852	
SymSpan		0.751	
Digit span			0.772

Factor loading coefficients below 0.40 are not displayed.

Factor 1 with factor loading scores above 0.40 (i.e., LLAMA B, E, and F) was labelled as “LLAMA” as a single factor involving vocabulary learning, sound-symbol correspondence, and grammatical inferencing. Factor 2 (i.e., RotSpan and SymSpan) was labelled “visuospatial WM”. Factor 3 involved digit span, OSpan, and LLAMA D. While the LLAMA D as the measure of sound recognition and forward digit span for PSTM loaded onto factor 3 with no cross-loadings, OSpan as the measure of verbal complex WM cross-loaded onto factors 2 and 3 at above the 0.40 threshold. Following the suggestions on factor solutions with cross-loading items in social sciences (e.g., Costello & Osborn, 2005; Matsunaga, 2010), Ospan was kept in the model since at least two other variables loaded onto each factor other than the cross-loading Ospan. Therefore, the factor 3 with the OSpan, digit span and LLAMA D was labelled “verbal-phonological memory”.

The three factors from the EFA were saved as variables using the regression method. To address the second research question regarding the relationship between L3 experience and the cognitive factors, analyses of variance with the L3 experience as the independent variable and the three factors as the dependent variables were conducted. The relationship between L3 experience representing the number of semesters studying the L3 course in a row and the cognitive factors were explored by bivariate correlations and linear regressions with the L3 experience as the predictor factor and the three cognitive factors as the outcome variables. (see Appendix E for the correlations and regression analyses). All of the three correlations lacked significance. Accordingly, linear prediction models of regression were not significant,, indicating a dissociation between the L3 domain experience and the cognitive factor variables.

To address the third research question regarding the predictive power of the LA and WM measures on the L3 listening and reading comprehension as outcome variables, hierarchical regression analyses were conducted. The descriptive statistics and bivariate correlation matrices for the hierarchical regression analyses for both outcome variables are presented in Appendix F. In the regression analyses, L3 experience was entered at step one in the models to control for the effect of overall L3 experience on the L3 comprehension scores, while the three cognitive factors were entered simultaneously at step two to test their predictive power in the L3 comprehension scores beyond the L3 experience.

L3 experience as the control variable had a significant bivariate correlations with the L3 listening and reading comprehension scores while the cognitive factors did not demonstrate any significant correlations.

A statistically significant hierarchical regression model was obtained predicting L3 listening comprehension as shown in Table 4. As signalled in the bivariate correlations, L3 experience was a significant predictor, explaining 38% of the variance in L3 listening comprehension in step one. However, the cognitive variables at step two did not contribute any significant change in the variance explained beyond L3 experience. Similarly, L3 experience was a significant predictor, explaining 14% of variance in L3 reading comprehension at step one. However, the cognitive variables did not contribute significantly to the variance explained beyond L3 experience at step 2as shown in Table 5.

**Table 4:** Hierarchical regression predicting L3 listening comprehension

Steps		Unstandardized coefficients		Standardized coefficients				
	Predictors	B	SE	Β	t	F	*R* ^2^	ΔR^2^
1	Constant	33.21	5.50		6.03***			
	L3 experience	5.5	1.26	0.62	4.38***	19.18***	0.38	
2	Constant	31.1	5.35		5.81***	7.45***	0.51	0.13
	L3 experience	5.98	1.96	0.67	5.00***			
	Factor 1 (LLAMA)	4.12	2.16	0.25	1.91			
	Factor 2 (visuospatial WM)	0.428	2.01	0.03	0.21			
	Factor 3 (verbal-phono. memory)	−4.06	2.07	− 0.27	−1.97			

***p <.001, **p<. 01, *p <. 05

**Table 5:** Hierarchical regression predicting L3 reading comprehension

Steps		Unstandardized coefficients		Standardized coefficients				
	Predictors	B	SE	Β	t	F	*R* ^2^	ΔR^2^
1	Constant	44.48	6.78		6.56***	5.12*		
	L3 experience	3.5	1.55	0.38	2.26*		0.14	
2	Constant	43.08	7.25		5.94***	1.6		
	L3 experience	3.79	1.62	0.408	2.34*		0.19	0.04
	Factor 1 (LLAMA)	2.44	2.92	0.14	0.83			
	Factor 2 (visuospatial WM)	0.74	2.72	0.05	0.27			
	Factor 3 (verbal-phono. memory)	−2.46	2.8	− 0.16	− 0.88			

***p <.001, **p<. 01, *p <. 05

In summary, the measures of LA and memory constitute three distinct factors: the LLAMA (i.e., LLAMA B, E, F), the visuospatial complex WM (SymSpan and RotSpan), and the verbal-phonological memory (OSpan, forward digit span, and LLAMA D). The L3 domain experience was not significantly related to these three factors of cognitive individual differences. L3 domain experience was the only significant predictor of L3 listening and reading comprehension, with no contribution of cognitive factors beyond L3 experience.

## Discussion

### Structural Relationship Between the Cognitive Factors

Our first research question aimed to explore the structural relationship between the LA and WM variables. The three-factor solution confirmed the distinction between the visuospatial WM and the LLAMA subtests. The visuospatial WM factor (i.e., RotSpan and SymSpan) involved domain-general non-linguistic cognitive abilities. This factor could be interpreted as the variable to probe the role of a domain-general non-linguistic latent factor in L3 learning following the arguments in L2 learning about how domain-general or language-specific L2 learning proceeds in post-critical age individuals (e.g., DeKeyser, 2000; Skehan, 2019). The LLAMA factor (i.e., grammatical inferencing, rote memory, and sound-symbol correspondence) also conformed to previous research reporting LLAMA B and F to load together under the LA factor (e.g., Granena, 2013; Xiang et al., 2012; Yalçın et al., 2016). Winke (2013) also reported that these two subtests were the variables to load most strongly to the LA latent factor.

The overlapping point between the LA and WM factors was observed in the verbal memory tasks, i.e., OSpan, PSTM, and LLAMA D. The verbal storage aspect of the LLAMA D sound recognition subtest can be the aspect to connect to the PSTM and verbal WM in the present study. Several validity studies examined the essence of the LLAMA D as an implicit test of LA. Granena’s (2013) observation that LLAMA D loading onto the same factor as serial reaction time measuring implicit learning as opposed to the other three LLAMA subtests was interpreted as LLAMA D representing an implicit aspect of LA with a lower level of conscious attention while the other three subtests function at a conscious attention level tapping explicit LA. Saito (2017) particularly adjusted the task instructions to guide the participants to check the sound during the initial exposure to the target nonwords to distract the participants’ conscious attention from the task and tap the unintentional ability to perceive and recognise sounds from among familiar and unfamiliar non-words in the LLAMA D. Saito found a similar distinction in the correlation patterns with oral L2 abilities between LLAMA D and the other three subtests similar to Granena’s (2013) findings. Suzuki (2021) further probed Saito’s sound check design adding response time and confidence rating to probe the implicitness of LLAMA D, observing that confidence ratings and response times could have a higher potential to represent an implicit aspect of LA rather than the accuracy scores as the outcome measure. Iizuka and DeKeyser (2023) compared the different task instructions (i.e., “memorisation” with explicit attention in the LLAMA D manual, “just listen” in Granena, 2013, and “sound check” in Saito, 2017) and observed that serial reaction time was a significant predictor of LLAMA D only in the “just listen” condition. This finding confirmed Granena’s observation that the serial reaction time measure was closely related to the LLAMA D when the instructions did not signal memorisation with explicit attention in the test phase. However, the “sound check” condition did not necessarily confirm the relationship to serial reaction time measuring implicit learning, leaving an ambiguity to its validity as a measure of implicit LA even in implicit instruction conditions.

The present study implemented LLAMA D v.3 with updates in the presentation of the target sounds. This version did not separate the target words from the test item and presented them in a continuous list. The participants were instructed to listen to the sounds and click the “repeated word” button if they thought they heard that sound since the start of the task and click the “new word” button otherwise. The factor analysis indicated that LLAMA D v.3 loaded onto the same factor as the digit span and OSpan, which involve conscious attention for verbal storage and processing. Unsworth and Engle (2005) particularly observed that WM differences in OSpan were larger under intentional rather than incidental learning conditions. Therefore, the other two tasks representing the verbal-phonological factor in this study reside closer to the explicit end of the implicit/explicit continuum. LLAMA D was observed to load with these two more explicit verbal tests, implying that it did not represent a structurally distinct latent variable outside of focal attention as opposed to the other tasks.

The verbal-phonological factor in the present study was most strongly represented by PSTM, highlighting the phonological storage capacity. In the validity study of the Hi-LAB test battery, PSTM (measured with a letter span task) was one of the significant predictors of advanced-level L2 listening and reading comprehension along with available long-term memory and implicit learning (Linck et al., 2013). In this study, the verbal-phonological factor involved the complex OSpan with storage and processing tasks. It is worth discussing the latent variable that the OSpan is likely to represent. Foster et al. (2015) reported that the full block version of the OSpan displayed lower predictive power on the general intelligence as opposed to the visuospatial complex WM tasks, drawing attention to the use of OSpan as a complex WM measure on its own as a non-linguistic verbal WM measure.

### Relationship Between L3 Experience and Cognitive Variables

Our second research question explored the relationship between L3 experience and the cognitive variables derived as factors from EFA. The L3 experience in this study was operationalised as the number of additional L3 courses taken in a row increasing in level at university, rather than a direct measurement of L3 linguistic knowledge or proficiency due to the different L3s and lack of comparable proficiency measures in all the included L3s. The bivariate correlations between L3 experience and the cognitive factors did not show any statistically significant correlations, as L3 experience did not explain any significant variance as a predictor factor in the cognitive factors. At this point, it is crucial to note the definitions of multilingualism and how it is represented in the background literature in discussing the relationship between multilingualism and cognitive factors. For example, Espi-Sanchis and Cockcroft (2022) particularly measure the “balance” of proficiency both within the language skills (i.e., speaking, reading, and comprehension) and between the three languages of their young adult participants in South Africa, speaking a wide range of the local languages as well as English in varying orders of acquisition (e.g., English was reported as L1, L2 or L3 by different participants). This within and between-language balance of language were computed as two separate variables based on the participants’ self-ratings on the Language Experience and Proficiency Questionnaire (LEAP-Q; Marian et al., 2007). The verbal and visuospatial storage and processing variables were regressed by the within and between-language balance variables, indicating a relationship only between verbal processing and between-language balance. Espi-Sanchis and Cockcroft proposed that the balance of language proficiency across the participants’ different languages can be a confounding factor for the potential effect of multilingualism experience onWM with young adult participants.

Bouffier et al. (2020) also investigated multilingualism and cognitive advantages question with trilingual young adult participants in Luxembourg measuring language proficiency in all three languages with vocabulary tests and exploring its relationship with auditory-verbal and visuospatial WM and attention measures. They observed that only non-native language proficiency was related to auditory-verbal WM, while attention measures and L1 proficiency all remained dissociated. While this variation emphasizes the significance of contextual and sample-related particularities across research designs in observing and measuring multilingualism, the relationship of multilingualism with different cognitive constructs also appears differentiated, also highlighting the roles of SES, balance of proficiency, frequency and context of language use in characterising the populations and research contexts.

Studies observing multilingualism in classroom contexts in young adults also represent multilingualism in varying ways. Huang et al. (2021) compare two groups of language majors at a university in China on their LA and WM scores, exploring the effect of language learning experience. The two groups differed in that one group studied only L2 English, while the other simultaneously learned various L3s along with the L2. They observed that the trilingual group significantly outperformed the bilingual group in LLAMA D, the phonological coding subtest of LLAMA D, which is also significantly predicted by fractal 2-back task of WM. This finding was further explored in a mediation analysis, indicating a significant mediating role of WM in the relationship between LLAMA and language experience. Similarly, Huang, Loerts, and Steinkrauss (2022) compared an L2 English only and L2 English + L3 learning group at a language major program in China, measuring LA and OSpan for WM. These groups were matched for L2 English proficiency, while multilingualism was represented as a categorical variable, with the simultaneous L3 learning as the distinguishing aspect. In pre-test/post-test design with the LLAMA and OSpan measures implemented before and after a semester, the learning effects in OSpan was observed to be significant in the L2 + L3 group than the L2 only group, taken as evidence for the effect of multilingual learning intensity by language on cognitive development. In a similar setting with language majors at a university in China, Ma et al. (2022) adopt a time related basis to create groups of different multilingualism experience (freshman, sophomore, and junior groups representing language proficiency) with either L2 or L2 + L3 training. They did not observe any significant differences between L2 and L3 groups in any subcategory of inhibition. However, further dividing the L3 into three groups by the length of L3 study indicated nuanced differentiation with a significantly higher performance of the L3 junior group (longer time of L3 learning) in response inhibition conditions. Our study adopted a length of L3 learning as the representation of multilingualism as a continuous variable representing the number of semesters in a rising level of proficiency in a particular L3, aiming to reflect multilingualism in a more sensitive metric level since the L3s were more diverse and L3 linguistic proficiency more variant across the sample The lack of a significant relationship between L3 experience and the cognitive factors represented by the subtests of LLAMA, visuospatial and verbal complex WM measures provides support for the null results observed in studies with young adult university students with varying levels of L3 learning experience. Considering the particularity of the cognitive abilities observed to differ by level of L3 experience such as in inhibition (Ma et al., 2022) or mediation effect of WM in the relationship of multilingual experience with LLAMA D only (Huang et al., 2021), it is worth highlighting that with mostly classroom instructed L3 learning experience of young adults at university levels, the relationship of multilingualism with cognitive variables can be manifested in more specific sub-abilities rather than more global domain-general abilities as measured by the complex WM tasks or phonological short term memory as our study presented.

### Cognitive Factors Predicting L3 Listening and Reading Comprehension

Our third research question probed the predictive power of the cognitive factors in explaining variance in L3 comprehension in a smaller subsample. The hierarchical regressions revealed that only L3 experience was a significant predictor of both listening and reading comprehension in L3 at the first step. The cognitive factors entered at the second step did not significantly contribute any additional variance to the prediction model beyond L3 experience.

At this point, it is worth noting that this research question could be addressed with only a smaller subsample due to the limitations in obtaining appropriate measures of L3 comprehension in different languages as L3s in the study. This requires caution in generalising the results on L3 comprehension. Therefore, we emphasize that our findings on L3 listening and reading comprehension represent a smaller subsample studying the L3s (i.e., German, Spanish, French, and Italian) that allowed measurement with proficiency tests following a common framework.

The present study aimed to test how the LA and WM variables that have been observed extensively in L2 outcomes further extends to L3 outcomes with listening and reading comprehension. To represent L3 outcomes, the two receptive skills were tested for text-level meaning making with varied response types such as multiple choice, short answer or matching at a global level to represent functional language skills. An enhanced ecological validity was also aimed by operationalising L3 performance beyond lexical and sentence level performance. Therefore, it becomes crucial to examine how L2 and L3/n outcomes were measured in LA and WM studies.

There is a larger accumulated body of research in L2 contexts representing language outcomes at comprehension level regarding LA and WM. For instance, Sok and Shin (2022) observed L2 listening comprehension to relate with LA through mediation of metacognitive awareness of listening strategies in adolescents with a mean age of 12.48 years with L2 English instruction at school for three years in South Korea. Similarly, Sparks et al. (2011) conducted a longitudinal study with high school students starting at grade 1 (mean age 6.9 years) through grade 10 (mean age 16.4 years) in the US. The participants had experienced average two years of instruction in one of Spanish, German, or French as L2 at the end of grade 10. The participants completed a large battery of tests including the MLAT for LA and L2 listening and reading comprehension. The reading comprehension was a letter in the L2 and measured through multiple choice items while L2 listening comprehension was measured integrated to speaking with interviews, where the recorded performances were scored for pronunciation, vocabulary, grammar, comprehensibility, and listening comprehension. The factors that included the LA subtests were not observed to significantly predict L2 listening comprehension. However, the factor structure including the spelling clues and phonetic script subtest of the MLAT significantly explained 11% of the variance in L2 reading. These subtests tap abilities that include noticing connections between phonemes and orthography, similar to the LLAMA E subtest tapping phoneme-grapheme correspondence. The factor structure included other tests of spelling, representing a more complex factor of written and auditory correspondence ability both in L1 and L2, rather than a refined examination of subtests of LA.

The relationship of WM with L2 listening and reading comprehension is more extensive with varied representations of WM and comprehension. There is an overall consistency in the observation that WM is significantly correlated with L2 listening and reading comprehension (see Linck et al., 2014, for a review). However, to our knowledge, studies on cognitive individual factors in L3/n contexts have measured cognitive factors in non-verbal instruments and represented L3/n variable as a grouping factor in instructed settings (i.e., Ma et al., 2018; Huang et al., 2021, 2022), or as vocabulary knowledge (e.g., Bouffier et al., 2020; Guðmundsdóttir & Lesk, 2019), and self-reported proficiency or language use (e.g., Espi-Sanchis & Cockcroft, 2022) in more naturalistic settings. Therefore, it is difficult to compare our null results in explaining variance in L3 comprehension with cognitive factors of LA and WM in terms of the influence of cognitive factors in L3 outcomes. However, considering the findings from L2 research which present more nuanced and metacognition-mediated relationships between particularly LA and L2 outcomes, it is as a significant insight to consider including metacognition or cognitive awareness of mental processes when complex abilities such as comprehension are investigated with relation to cognitive individual factors. Moreover, the target population of university/college-level young adults, who already show overall higher performance in WM and inhibition tasks compared to children/adolescents (e.g., Park & Kim, 2024), might also manifest an enhanced impact of these high-level awareness factors. They also tend to form groups with homogeneous intellectual abilities and intensive experience of language instruction, who potentially become more likely to develop metacognition or strategies in performing across multiple languages. This might neutralise the effects of individual differences in cognitive factors such as LA and WM in L3/n learners. Likewise, research on L2 reading and WM relationship indicated some interactions with some factors such as topic familiarity and content knowledge (e.g., Alptekin & Erçetin, 2011; Leeser, 2007). Their findings could relate to our above interpretation of participants’ background resources in foreign language reading comprehension. L3 reading skills in a developing language could require a design with more control over potentially mediating factors such as task conditions (i.e., literal or inferential texts), topic familiarity, strategy use, or content knowledge. Hummel (2009) reported a more specific significant relationship between grammatical inferencing and L2 reading comprehension in young adults while the phonological memory was not significantly related, implying a more segmented and specified relationship between the complex skill of reading and the particular LA abilities.

To summarise, our study confirmed the distinction between LA (LLAMA B, E, and F v.3) and the visuospatial WM (i.e., RotSpan and SymSpan). The potential overlap between these two constructs appeared in the verbal-phonological memory abilities (i.e., digit span, OSpan, and LLAMA D). The L3 experience was not significantly related to any of these cognitive factors. The cognitive factors did not manifest any predictive roles on L3 listening or reading comprehension beyond L3 experience.

## Conclusions

The present study highlighted the ambiguity between the LA and WM structures to reside in the verbal-phonological memory abilities tapping PSTM, the non-linguistic verbal measure of OSpan, and the sound recognition subtest of LLAMA D v.3. As common measures of LA and WM in many second/foreign language learning studies, it is essential to clarify the latent variables these instruments represent with systematic validation studies. Operationalised as the time spent taking classroom instruction in the additional L3, L3 experience did not significantly correlate with the LA and WM factors. The predictive roles of the LA and WM factors beyond L3 experience on L3 listening and reading comprehension were also non-significant. It is worth noting that cognitive individual differences investigated with college/university students with high intellectual skills and less variation within the sample might increase the likelihood of a neutralising individual differences in the cognitive factors of LA and WM through metacognition or strategy use. Testing these questions with similar populations for multilingualism effects might require designs including measures of metacognition, linguistic or strategic awareness due to enhanced experience of multilingualism.

### Limitations and Implications

Convenience sampling was adopted in this study. Participation was voluntary, potentially attracting participants with higher motivation about their L3 learning experience. The data collection period was under post-COVID-19 conditions, potentially influencing the participants’ overall motivation and willingness to attend physical sessions for research purposes. Additionally, the sample was homogeneous in terms of L1 and L2 background as well as general intellectual and cognitive abilities, displaying the general bias of the cognitive research studies to frequently employ university students due to convenience.

This study implemented the updated version 3 of the LLAMA and the shortened versions of the RotSpan, SymSpan, and OSpan. The findings highlighted the crossing point of LLAMA D, OSpan, and the digit span tasks, requiring closer attention to latent factors in the arguments of LA and WM relationships.

It may be beneficial to emphasize the operationalization of language experience and influential variabilities in participants’ backgrounds. This study operationalised language experience as the number of L3 courses taken in a row rising in proficiency. However, a directly linguistic measure such as vocabulary tests comparable across the L3s as well as control groups with lower L2 proficiency could have provided a more robust and nuanced picture of the cognitive factors through the continuum of L3 proficiency. This approach allowed representations of different L3 learning experiences; however, more variability in L1 and L2 proficiencies, SES or language balance within individuals also introduced potential areas of variation. Therefore, it is important to clarify and control them for the generalisability of multilingual studies.

## Supplementary Information

Below is the link to the electronic supplementary material.

Supplementary material 1 (DOCX 16.6 kb)

Supplementary material 2 (DOCX 13.3 kb)

Supplementary material 3 (DOCX 18.3 kb)

Supplementary material 4 (DOCX 13.9 kb)

Supplementary material 5 (DOCX 18.1 kb)

Supplementary material 6 (DOCX 19.3 kb)

## Data Availability

The datasets generated by the research and analyzed during the current study are available in the Open Science Framework (OSF) repository, https://osf.io/8c39x/?view_only=a27a983d20c74698b374e829f6246b05.
