# Supplementary material for: Cognitive Individual Differences in Multilingualism: Language Aptitude and Working Memory in L3 Learners
Source: J Psycholinguist Res. 2026 Jul 1;55(4):97. doi: 10.1007/s10936-026-10268-3 (PMC13323783; doi:10.1007/s10936-026-10268-3)
Supplement: Supplementary file 1 — Supplementary material 1 (DOCX 16.6 kb) [file 10936_2026_10268_MOESM1_ESM.docx]

Appendix A. Frequencies across L3 domain experience and additional foreign languages

| Participant Profile | *N* | Percent |
| --- | --- | --- |
| Number of semesters at the time of data collection |  |  |
| 2 (Freshman) | 1 | .9 |
| 3 – 4 (Sophomore) | 40 | 36.7 |
| 5 – 6 (Junior) | 37 | 34 |
| 7 – 12 (Senior) | 31 | 28.3 |
| Total | 109 | 100 |
| Levels of L3 courses taken | *N* | Percent |
| 0 (L2-only) | 24 | 22.2 |
| 1 course (Elementary, 101 level) | 16 | 14.8 |
| 2 courses (Elementary, 102 level) | 25 | 23.1 |
| 3 courses (Intermediate, 201 level) | 12 | 11.1 |
| 4 courses (Intermediate, 202 level) | 13 | 12 |
| 5 courses (Upper intermediate-advanced, 301 level) | 9 | 8.3 |
| 6 courses (Upper intermediate-advanced, 302 level) | 6 | 5.6 |
| 7 courses (Advanced or literature coursess) | 2 | 1.9 |
| 8 courses (Advanced or literature coursess) | 1 | .9 |
| Total | 108 | 99.1 |
| Missing | 1 | .9 |
| Total | 109 | 100 |
| The distribution of L3 languages | *N* | Percent |
| Arabic | 1 | .9 |
| Chinese | 6 | 5.5 |
| French | 23 | 21.1 |
| German | 10 | 9.2 |
| Italian | 1 | .9 |
| Japanese | 3 | 2.8 |
| Korean | 11 | 10.1 |
| Portuguese | 8 | 7.3 |
| Russian | 4 | 3.7 |
| Spanish | 18 | 16.5 |
| L2-only (No L3) | 24 | 22 |
| Total | 109 | 100 |
