# Supplementary material for: Cognitive Individual Differences in Multilingualism: Language Aptitude and Working Memory in L3 Learners
Source: J Psycholinguist Res. 2026 Jul 1;55(4):97. doi: 10.1007/s10936-026-10268-3 (PMC13323783; doi:10.1007/s10936-026-10268-3)
Supplement: Supplementary file 2 — Supplementary material 2 (DOCX 13.3 kb) [file 10936_2026_10268_MOESM2_ESM.docx]

| Languages as L3s | *N* | Percent | Mean (SEs) L3 experience (semesters of L3 courses taken) |
| --- | --- | --- | --- |
| Arabic | 1 | .9 | NA* |
| Chinese | 6 | 5.5 | 2.5 (.76) |
| French | 23 | 21.1 | 2.96 (.36) |
| German | 10 | 9.2 | 3.78 (.59) |
| Italian | 1 | .9 | NA* |
| Japanese | 3 | 2.8 | 3.33 (1.45) |
| Korean | 11 | 10.1 | 2.91 (.51) |
| Portuguese | 8 | 7.3 | 1.75 (.16) |
| Russian | 4 | 3.7 | 2 (.71) |
| Spanish | 18 | 16.5 | 3.89 (.40) |
| L2-only (No L3) | 24 | 22 | 0 (.00) |
| Total | 109 | 100 |  |
| **Note.* For Arabic and Italian, mean and standard errors were not computed since L3 experience values were constant, coming from one participant who studied these languages as L3s. The level of L3 experience was two semesters for Arabic, and four semesters for Italian. | | | |

Appendix B. Means (and standard errors) of L3 experience by languages
