# Supplementary material for: Cognitive Individual Differences in Multilingualism: Language Aptitude and Working Memory in L3 Learners
Source: J Psycholinguist Res. 2026 Jul 1;55(4):97. doi: 10.1007/s10936-026-10268-3 (PMC13323783; doi:10.1007/s10936-026-10268-3)
Supplement: Supplementary file 3 — Supplementary material 3 (DOCX 18.3 kb) [file 10936_2026_10268_MOESM3_ESM.docx]

Appendix C. L3 comprehension tests in the versions administered in the study

| Language test | Listening | Total items | Reading | Total items |
| --- | --- | --- | --- | --- |
| German^[[1]](#endnote-1)^  (ZERTIFIKAT B1 of Goethe Institute) | Part 1: Five short listening texts, 10 items  Part 2: One longer text, 5 MC items  Part 3: One longer text, 7 True-False items  Part 4: One discussion, matching statements with speakers in 8 items | 30 | Part 1: One text, 6 True-False items  Part 2: Two texts, 6 MC items  Part 3: Matching situations with announcements, 7 items  Part 4: Matching paragraphs with people’s opinions, 7 items  Part 5: One short text with 5 MC items | 30 |
| Spanish^[[2]](#endnote-2)^ (DELE, Level B1) | Part 1: Five short listening texts, 5 MC items  Part 2: One longer text, 6 MC items  Part 3: Six short texts, 6 MC items  Part 4: Six short texts, 6 matching items  Part 5: One conversation, matching statements with speakers in 6 items | 30 | Part 1: Six short texts and personal introductions, 6 matching items  Part 2: One longer text, 6 MC items  Part 3: Matching paragraphs with people’s backgrounds, 6 items  Part 4: Insert missing sentences into a longer text, 6 items  Part 5: A cloze test with 6 MC items | 30 |
| French^[[3]](#endnote-3)^ (DELF, Level B1) | Part 1: One text, 2 short-answer items, 4 MC items  Part 2: One text, 3 short-answer items, 3 MC items  Part 3: One text, 3 short-answer items, 5 MC items | 20 | Part 1: One text, 10 True-False items  Part 2: One text, 5 MC items, 6 open-ended items | 21 |
| Italian^[[4]](#endnote-4)^ (CILS, Level B1) | Part 1: Seven short listening texts, 7 MC items  Part 2: One longer dialogue, 7 MC items  Part 3: One longer text, choosing statements mentioned in the text among others not mentioned, 13 items | 27 | Part 1: One text, 7 MC items  Part 2: One text, selecting statements mentioned in the text among those not mentioned, 15 items  Part 3: Putting a paragraph in order from scrambled sentences, 10 items | 32 |

1. Available on 7 Feb 2026 at: https://www.goethe.de/pro/relaunch/prf/materialien/B1/b1_modellsatz_erwachsene.pdf [↑](#endnote-ref-1)
2. Available on 7 Feb 2026 at: <https://examenes.cervantes.es/es/dele/preparar-prueba?_gl=1*kr9unz*cent_ga*MTg4OTk1MjUzMy4xNzA5NTc3MDE1*cent_ga_57JPCLFY9Z*MTcwOTU3NzAxNC4xLjAuMTcwOTU3NzAxNC42MC4wLjA.*cent_ga_5NVGDGFZ4P*MTcwOTU3NzAxNC4xLjAuMTcwOTU3NzAxNC42MC4wLjA.*cent_ga_VD16MRRKY3*MTcwOTU3NzAxNC4xLjAuMTcwOTU3NzAxNC42MC4wLjA>. [↑](#endnote-ref-2)
3. Available on 7 Feb 2026 at: https://learnfrenchdelf.com/delf-b1/sample-papers/set-1/ [↑](#endnote-ref-3)
4. Available on 7 Feb 2026 at: https://cils.unistrasi.it/public/articoli/197/Files/quaderno_uno-b1_giugno_2012.pdf [↑](#endnote-ref-4)
