# Supplementary material for: Cognitive Individual Differences in Multilingualism: Language Aptitude and Working Memory in L3 Learners
Source: J Psycholinguist Res. 2026 Jul 1;55(4):97. doi: 10.1007/s10936-026-10268-3 (PMC13323783; doi:10.1007/s10936-026-10268-3)
Supplement: Supplementary file 4 — Supplementary material 4 (DOCX 13.9 kb) [file 10936_2026_10268_MOESM4_ESM.docx]

Appendix D. Descriptive statistics for the explaratory factor analysis

|  | *Mean* | *SD* | *N* | Missing *N* |
| --- | --- | --- | --- | --- |
| LLAMA B | 9.28 | 4.387 | 107 | 0 |
| LLAMA D | 11.25 | 2.911 | 107 | 0 |
| LLAMA E | 11.28 | 5.080 | 107 | 0 |
| LLAMA F | 12.10 | 3.613 | 107 | 0 |
| OSpan* | .595 | .368 | 106 | 1 |
| RotSpan | 8.96 | 3.062 | 104 | 3 |
| SymSpan* | 3.601 | 1.061 | 106 | 1 |
| Digit span | 7.10 | 1.042 | 107 | 0 |
| *Note*. Pairwise deletion of missing values applied.  *Versions with data transformations implemented after data cleaning for univariate normality. | | | | |
