# Supplementary material for: Cognitive Individual Differences in Multilingualism: Language Aptitude and Working Memory in L3 Learners
Source: J Psycholinguist Res. 2026 Jul 1;55(4):97. doi: 10.1007/s10936-026-10268-3 (PMC13323783; doi:10.1007/s10936-026-10268-3)
Supplement: Supplementary file 5 — Supplementary material 5 (DOCX 18.1 kb) [file 10936_2026_10268_MOESM5_ESM.docx]

Appendix E. L3 domain experience predicting the cognitive factor variables

Table 1. Descriptive statistics for L3 experience and the cognitive factors

|  | Mean | Std. Dev. | N |
| --- | --- | --- | --- |
| L3 experience | 2.44 | 2.003 | 103 |
| Factor 1 (LLAMA) | .010 | .999 | 103 |
| Factor 2 (visuospatial WM) | .009 | .999 | 103 |
| Factor 3 (verbal-phono. memory) | -.005 | 1.003 | 103 |

Table 2. Bivariate correlations between L3 experience and the cognitive factors

|  | Factor 1 (LLAMA) | Factor 2 (visuospatial WM) | Factor 3 (verbal-phono. memory) |
| --- | --- | --- | --- |
| L3 experience | .166 | .133 | .002 |
| Note. *N*=103, listwise deletion of missing values applied. All *p*s > .05. | | | |

Table 3. Simple linear regressions with L3 experience as predictor and the cognitive factors as outcome variables

|  |  | Unstandardized coefficients | | Standardized coefficients |  |  |  |  |
| --- | --- | --- | --- | --- | --- | --- | --- | --- |
| Outcome Variable | Predictors | *B* | *SE* | *Β* | *t* | *F* | *p* | *R^2^* |
| Factor 1 (LLAMA) | Constant | -.131 | .155 |  | -.842 |  |  |  |
|  | L3 experience | .058 | .049 | .116 | 1.175 | 1.381 | .243 | .013 |
| Factor 2 (visuospatial WM) | Constant | -.151 | .155 |  | -.997 |  |  |  |
|  | L3 experience | .066 | .049 | .133 | 1.345 | 1.809 | .182 | .018 |
| Factor 3 (verbal-phono. memory) | Constant | -.008 | .157 |  | -.050 |  |  |  |
|  | L3 experience | .001 | .050 | .002 | .022 | .000 | .982 |  |
| *Note.* *N* = 103, listwise deletion of missing values applied. | | | | | | | | |
