# Supplementary material for: Cognitive Individual Differences in Multilingualism: Language Aptitude and Working Memory in L3 Learners
Source: J Psycholinguist Res. 2026 Jul 1;55(4):97. doi: 10.1007/s10936-026-10268-3 (PMC13323783; doi:10.1007/s10936-026-10268-3)
Supplement: Supplementary file 6 — Supplementary material 6 (DOCX 19.3 kb) [file 10936_2026_10268_MOESM6_ESM.docx]

Appendix F. Descriptive statistics and bivariate correlations between the predictor variables and L3 comprehension scores

L3 Listening Comprehension Scores

Table 1. Descriptive statistics for L3 listening comprehension and the predictor variables

|  | Mean | Std. Dev. | N |
| --- | --- | --- | --- |
| L3 listening comprehension | 55.53 | 14.91 | 33 |
| L3 experience | 4.06 | 1.68 | 33 |
| Factor 1 (LLAMA) | -.137 | .91 | 33 |
| Factor 2 (visuospatial WM) | .256 | 1.00 | 33 |
| Factor 3 (verbal-phono. memory) | -.149 | .99 | 33 |
| Note. *N* = 33, listwise deletion of missing values applied. | | | |

Table 2. The correlations between the L3 listening comprehension and the predictor variables

|  | 1 | 2 | 3 | 4 | 5 |
| --- | --- | --- | --- | --- | --- |
| 1. L3 listening comprehension | 1 |  |  |  |  |
| 2. L3 experience | .618*** | 1 |  |  |  |
| 3. Factor 1 (LLAMA) | .257 | .003 | 1 |  |  |
| 4. Factor 2 (visuospatial WM) | -.014 | .011 | .039 | 1 |  |
| 5. Factor 3 (verbal-phono. memory) | -.131 | .204 | -.013 | .221 | 1 |
| *Note*. ****p* <.001 | | | | | |

L3 Reading Comprehension Scores

Table 3. Descriptive statistics for L3 listening comprehension and the predictor variables

|  | Mean | Std. Dev. | N |
| --- | --- | --- | --- |
| L3 reading comprehension | 58.69 | 15.57 | 33 |
| L3 experience | 4.06 | 1.68 | 33 |
| Factor 1 (LLAMA) | -.137 | .91 | 33 |
| Factor 2 (visuospatial WM) | .256 | 1.00 | 33 |
| Factor 3 (verbal-phono. memory) | -.149 | .99 | 33 |
| Note. *N* = 33, listwise deletion of missing values applied. | | | |

Table 4. The correlations between the L3 reading comprehension and the predictor variables

|  | 1 | 2 | 3 | 4 | 5 |
| --- | --- | --- | --- | --- | --- |
| 1. L3 reading comprehension | 1 |  |  |  |  |
| 2. L3 experience | .377* | 1 |  |  |  |
| 3. Factor 1 (LLAMA) | .148 | .003 | 1 |  |  |
| 4. Factor 2 (visuospatial WM) | .023 | .011 | .039 | 1 |  |
| 5. Factor 3 (verbal-phono. memory) | -.065 | .204 | -.013 | .221 | 1 |
| *Note*. **p* <.05 |  |  |  |  |  |
